# Comment on “The Association of Hypertension Based on Systolic and Diastolic Blood Pressure With Dental Visits in the Population Aged 45 and Older: Cross‐Section Study Results From the China Health and Retirement Longitudinal Study”

**DOI:** 10.1002/clc.70442

**Published:** 2026-08-07

**Authors:** Zhe Zhang, Dong Cai, Suhua Xu

**Affiliations:** ^1^ College of Traditional Chinese Medicine Fujian University of Traditional Chinese Medicine Fujian Fuzhou China

Dear Editor,

We read with interest the study by Ao et al., which examined the associations of systolic and diastolic blood pressure and hypertension status with dental visits among Chinese adults aged 45 years and older [1]. By analyzing measured blood pressure together with questionnaire data from the China Health and Retirement Longitudinal Study, the authors provide useful population‐based evidence on patterns of dental attendance among middle‐aged and older adults. However, one interpretive issue warrants clarification: the manuscript alternately describes the measured outcome as dental visits, oral disease, and oral health issues, although these terms do not represent the same construct.

The outcome was determined by whether participants had visited a dentist during the previous year for dental care, including dentures. The proportions reporting dental visits were 79.82%, 82.05%, and 84.25% in the normal blood pressure, prehypertension, and hypertension groups, respectively. In the fully adjusted model, hypertension was associated with dental attendance with an odds ratio of 1.30 (95% confidence interval, 1.06–1.60) [1]. These findings quantify differences in dental attendance, but they do not provide corresponding estimates of the prevalence or severity of oral disease.

Importantly, this issue is not merely a limitation of self‐reported measurement. It reflects a difference in the underlying construct being assessed. Dental attendance measures dental healthcare utilization, whereas oral disease burden concerns the presence and severity of conditions such as dental caries, periodontal disease, and tooth loss. Dental visits may be driven by oral symptoms, but they may also reflect preventive examinations, restorative treatment, denture maintenance, healthcare access, or greater health awareness. Therefore, a higher probability of dental attendance among individuals with hypertension does not necessarily indicate a greater burden of oral disease. Establishing such a conclusion would require disease‐specific indicators, including standardized oral examinations, periodontal probing depth, clinical attachment loss, or tooth‐loss measures [2, 3].

We suggest that the authors consistently describe the outcome as dental visits or dental healthcare utilization throughout the abstract, results, discussion, and conclusion. It would also be helpful to clarify that dental attendance cannot serve as a direct surrogate for clinically assessed oral disease. If information on the reasons for attendance is available, distinguishing preventive, symptom‐driven, restorative, and denture‐related visits may further improve interpretation.

This clarification would not diminish the value of the study. Rather, it would more accurately define the scope of the findings and provide a clearer basis for future research that combines dental healthcare utilization data with clinical oral assessments to examine the relationship between hypertension and clinically assessed oral disease burden.

## Author Contributions

**Zhe Zhang, Dong Cai**, and **Suhua Xu:** conceptualization, methodology, writing–original draft, writing–review and editing.

## Funding

The authors have nothing to report.

## Ethics Statement

The authors have nothing to report.

## Consent

The authors have nothing to report.

## Conflicts of Interest

The authors declare no conflicts of interest.

## Data Availability

The authors have nothing to report.
